# Using Community-Impact Internships to Broaden AFU Commitment on Campus

**DOI:** 10.1093/geroni/igaa057.1806

**Published:** 2020-12-16

**Authors:** Andrea June, Carrie Andreoletti

**Affiliations:** Central Connecticut State University, New Britain, Connecticut, United States

## Abstract

Ongoing efforts to support Age Friendly University (AFU) principles must be campus-wide and include commitment from upper administration, faculty, staff, and students. This presentation will describe the leveraging of a new community-based collective partnership to develop a student internship model at Central Connecticut State University (CCSU) that could be used across campus to create a deeper investment in the AFU initiative. We will discuss the valuable collaboration with the Connecticut Healthy Living Collective, an emerging hub of state, regional and local agencies and organizations dedicated to healthy aging in communities throughout our state. The presenters will describe the process of establishing the Matter of Balance internship in psychological science with the support of our dean, knowing that it would eventually serve the broader AFU mission. We will highlight the value this intentional effort offers at multiple levels and how it might be replicated on other AFU campuses.

